# Pseudo-spontaneous nystagmus in lateral semicircular canal benign paroxysmal positional vertigo: Correlation with bow and lean test in a pitch plane

**DOI:** 10.1371/journal.pone.0242580

**Published:** 2020-11-19

**Authors:** Hyun-Jin Lee, Seong Ki Ahn, Chae Dong Yim, Dae Hwan Kim, Dong Gu Hur

**Affiliations:** 1 Department of Otorhinolaryngology, Gyeongsang National University Changwon Hospital, Gyeongsang National University College of Medicine, Changwon, Korea; 2 Department of Otorhinolaryngology, Gyeongsang National University Hospital, Gyeongsang National University College of Medicine, Jinju, Korea; 3 Institute of Health Sciences, Gyeongsang National University, Jinju, Korea; University College London, UNITED KINGDOM

## Abstract

**Objectives:**

We investigated the incidence and characteristics of pseudo-spontaneous nystagmus (PSN) in benign paroxysmal positional vertigo involving the lateral semicircular canal (LC-BPPV) and evaluated the correlation between PSN and the bow and lean test.

**Methods:**

We examined nystagmus in the sitting position using video-oculography goggles in 131 LC-BPPV patients. The positioning test and bow and lean test were also performed. Patients were divided into canalolithiasis and cupulolithiasis groups according to the character of nystagmus. In each group, the incidence and direction of PSN, correlation with the bow and lean test, and treatment outcome were analyzed.

**Results:**

PSN was observed in 25 cases (19.1%) in LC-BPPV patients, 7 of which were canalolithiasis and 18 of which were cupulolithiasis (*p* = 0.098). Of the 25 patients with PSN, 21 (84%) exhibited nystagmus consistent with the lean test whereas 4 (16%) exhibited nystagmus consistent with the bow test. In patients with PSN, nystagmus was observed in the bow and lean test in all cases (23/23), but in patients without PSN, no nystagmus was observed in 13 cases (13/87) in the bow and lean test (*p* = 0.048). The number of barbecue maneuvers performed until the end of treatment was 1.4 ± 0.7 in patients with PSN and 1.4 ± 0.9 in those without PSN (*p* = 0.976).

**Conclusion:**

We identified PSN in patients with LC-BPPV irrelevant of subtype. Moreover, all patients with PSN showed nystagmus in the bow and lean test. The direction of PSN was mostly consistent with that of the lean test (21/25, 84%). The presence of PSN was not related to the treatment outcome in this study.

## Introduction

Benign paroxysmal positional vertigo (BPPV) is the most common peripheral vestibular syndrome, which accounts for approximately 17–42% of all cases of vertigo [1]. It is characterized by sudden episodes of rapid-onset rotatory vertigo triggered by changes in head position with respect to the gravitational vector. The underlying pathogenic mechanism involves free-floating otoconial debris in the semicircular canal that moves within the canal (canalolithiasis) or debris near or that adheres to the cupula (cupulolithiasis), thereby exciting the ampulla. This stimulation produces abnormal vestibulo-ocular reflexes, resulting in vertigo and nystagmus that show different characteristics depending on the canal affected [2]. The most common BPPV variant is posterior canal (PC) BPPV, accounting for 60–90% of all BPPV cases, followed by lateral canal (LC) BPPV and anterior canal (AC) BPPV (5–30% and 1.2–12% of cases, respectively) [3–6].

Several recent studies have reported the occurrence of spontaneous nystagmus in the sitting position in patients with BPPV [7–10]. Such nystagmus is direction changing in nature, modulated by head position in the pitch plane. The diagnostic criteria for BPPV presented by the Barany Society in 2015 described the spontaneous nystagmus observed in LC-BPPV as pseudo-spontaneous nystagmus (PSN) [11]. In general, this nystagmus occurs due to otoconial debris affecting the deflection of the cupula through several mechanisms, because the head position is not at the null point of the pitch plane in the upright sitting position. PSN mainly appears in LC-BPPV and is observed in both canalolithiasis and cupulolithiasis. However, there are still several controversial factors related to PSN. Reports on the incidence of PSN vary widely, there are conflicting opinions on treatment outcome, and factors that affect the occurrence of PSN have not been clarified. Moreover, few studies have examined the association between PSN and the results of positioning tests or other nystagmus tests performed together to diagnose BPPV. Among these nystagmus tests, the bow and lean test has been utilized to determine the affected side in patients with LC-BPPV, by the direction of bowing nystagmus and leaning nystagmus instead of the intensity of nystagmus in the yaw plane [12]. Because the bow and lean test provides stimulation using the head position of the pitch plane rather than the yaw plane in the LC-BPPV patient, which is similar to the mechanism of PSN, it can be predicted that there will be some correlation between the two tests. Therefore, in the present study, we investigated the incidence and characteristics of PSN in patients with LC-BPPV, as well as the possible underlying mechanisms. In addition, the correlations between the direction and the incidence of nystagmus in PSN and in the bow and lean test were evaluated.

## Materials and methods

From January 2019 to January 2020, the medical records of 240 patients diagnosed with BPPV admitted to our outpatient clinic were retrospectively reviewed. Patients with history of head trauma, neurological or psychiatric disease, and otologic symptoms suggesting other inner ear diseases such as sudden sensorineural hearing loss, vestibular neuritis, labyrinthitis, and Ménière’s disease were excluded. We also excluded BPPV patients with multiple canal involvement. Among these 240 BPPV patients, 131 cases were LC-BPPV. There were 103 and 6 cases of PC- and AC-BPPV, respectively. PC- and AC-BPPV patients were excluded because there were no cases of PSN except in one patient with PC-BPPV. Thus, 131 patients diagnosed with LC-BPPV were included in the final analyses. With regards to the study, patients who fulfilled the inclusion criteria provided verbal consent at the time of recruitment. All patients were given a detailed explanation of the purpose of the study, the protocol, and any potential complications. Their data were recruited and fully anonymized. Ethical review (2020-03-043) was obtained from Gyeongsang National University Changwon Hospital, Institutional Review Board which stated that a written informed consent for a retrospective study is not required.

LC-BPPV was diagnosed using video-oculography goggles (VF405, Interacoustics, Denmark) to evaluate nystagmus without any effects of visual fixation. The presence of PSN was determined in the natural sitting position for more than 10 seconds without any head movement. Subsequently, the supine head roll and Dix-Hallpike tests were performed, followed by the bow and lean test in most cases. All enrolled patients reported repeated episodes of positional vertigo by rotating the head in the supine position, and typical direction-changing horizontal nystagmus was observed. When LC-BPPV was confirmed, the subtypes were classified as geotropic or apogeotropic nystagmus according to the direction of nystagmus during the supine roll test. The geotropic type was diagnosed as canalolithiasis with the lesion side toward the direction of strong nystagmus, and the apogeotropic type was diagnosed as cupulolithiasis with the lesion side toward the direction of weak nystagmus, considering the latency, duration, and null point of the nystagmus. When the difference in intensity of the nystagmus was unclear, the results of the bow and lean test were considered together to determine the affected ear. Patients were divided into the canalolithiasis group and the cupulolithiasis group to compare the incidence and direction of PSN. In patients who performed the bow and lean test together, the direction of nystagmus and the correlation with PSN were analyzed. In addition, caloric tests were performed in 18 cases with severe symptoms or difficulty obtaining a differential diagnosis.

Repositioning therapy for LC-BPPV patients was initiated immediately after diagnosis. All patients were treated with the barbecue maneuver. In patients with cupulolithiasis, the barbecue maneuver was performed after vibrating the mastoid area of the affected ear. Patients were followed up and tested at intervals of 1–3 days. When nystagmus was confirmed, the barbecue maneuver was repeated. If symptoms improved and the nystagmus disappeared completely, treatment was terminated. The treatment outcome was determined by the number of repeated barbecue maneuvers performed. No other treatment, such as administration of an anti-vertigo agent, was performed.

All statistical analyses were conducted using SPSS software for PC, version 21 (IBM, Corp., Armonk, NY, USA). Significant differences between groups were determined using the independent t test or chi-square test. All tests used a *p*-value < 0.05 as the cut-off for statistical significance.

## Results

**Fig 1:**
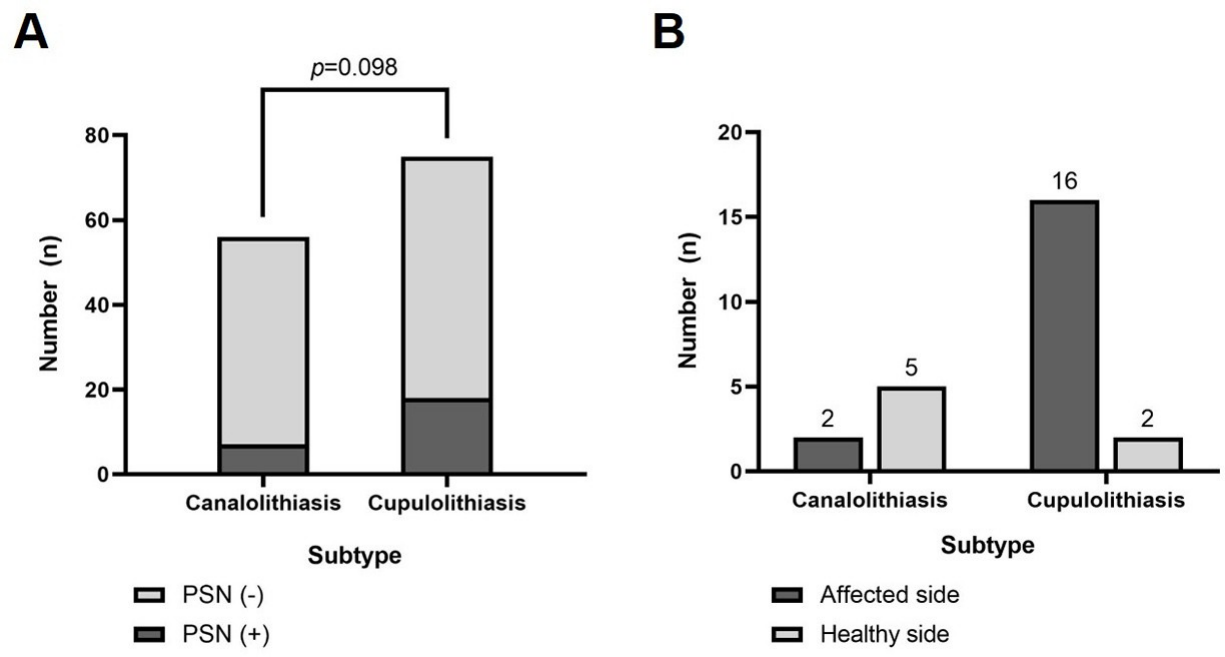
**(A)** Comparison of PSN incidence between canalolithiasis and cupulolithiasis in LC-BPPV patients. Of the 131 LC-BPPV patients, 25 (19.1%) showed PSN in the sitting position with the head perpendicular to the horizontal plane. When divided into subtypes, 7 patients (12.5%) had canalolithiasis and 18 (24%) had cupulolithiasis, and there were no statistically significant differences between the two groups (*p* = 0.098). **(B)** Comparison of PSN direction between canalolithiasis and cupulolithiasis in LC-BPPV patients. In the canalolithiasis group, PSN was more likely to be on the healthy side. By contrast, PSN was more often directed toward the affected side in the cupulolithiasis group. However, both directions of PSN occurred in all subtypes. PSN: pseudo-spontaneous nystagmus; LC-BPPV: lateral semicircular canal benign paroxysmal positional vertigo.

Of the 131 LC-BPPV patients, the male-to-female ratio was 45:86 and the average age was 55.5 ± 13.0 years; 25 patients (19.1%) had PSN. There were no significant differences in clinical characteristics between patients with and without PSN (Table 1). The LC-BPPV patients were divided into two groups according to their vertigo subtype, with 56 patients in the canalolithiasis group and 75 patients in the cupulolithiasis group. There were no significant differences in the ratios of age, sex, duration, and recurrence between the two groups (Table 2). The incidence of PSN observed in each group was 7 (12.5%) in the canalolithiasis group and 18 (24%) in the cupulolithiasis group, and these ratios were not significantly different (*p* = 0.098) (Fig 1A). In the PSN-positive patients, the direction of PSN was toward the affected side in 2 cases and toward the healthy side in 5 cases in the canalolithiasis group, whereas it was toward the affected side in 16 cases and toward the healthy side in 2 cases in the cupulolithiasis group (Fig 1B).

**Table 1:** Comparison of clinical characteristics according to the presence of PSN in LC-BPPV patients.

	PSN (+) (n = 25)	PSN (-) (n = 106)	*p*-value
Age (years)	60.6±10.7	54.4±13.2	0.321
Sex (male: female)	9:16	36:70	0.848
Affected side (right: left)	8:17	42:64	0.484
Subtype (canalolithiasis: cupulolithiasis)	7:18	49:57	0.099
Duration (days)	4.7±7.8	6.3±11.6	0.517
Recurred case	4/25	18/106	0.907
CRT	1.4±0.7	1.4±0.9	0.976

PSN: pseudo-spontaneous nystagmus; LC-BPPV: lateral semicircular canal benign paroxysmal positional vertigo; CRT: canalith repositioning therapy.

**Table 2:** Demographic findings between the canalolithiasis group and cupulolithiasis group in LC-BPPV patients.

	Canalolithiasis (n = 56)	Cupulolithiasis (n = 75)	*p*-value
Age (years)	54.2±11.5	56.5±14.0	0.316
Sex (male: female)	18:38	27:48	0.649
Affected side (right: left)	22:34	28:47	0.822
Duration (days)	6.4±12.6	5.6±9.5	0.693
Recurred case	10/56	12/75	0.781
CRT	1.4±0.7	1.4±0.9	0.578

LC-BPPV: lateral semicircular canal benign paroxysmal positional vertigo; CRT: canalith repositioning therapy.

**Fig 2:**
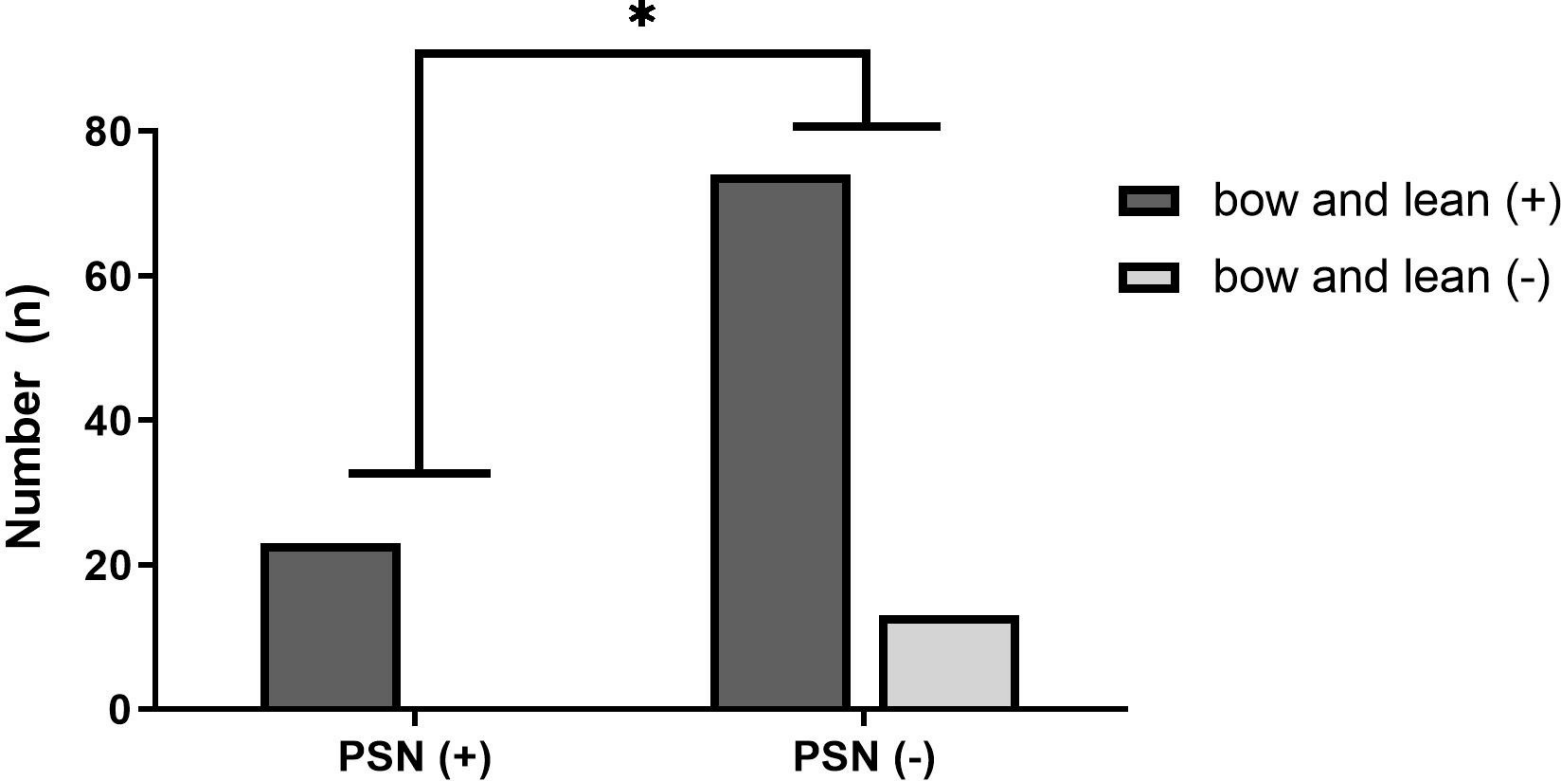
Comparison of the incidence of the bow and lean test in PSN-positive and PSN-negative groups. In the PSN-positive group, all patients had positive bow and lean tests (23/23). In the PSN-negative group, 13 of 87 patients (14.9%) did not show nystagmus in the bow and lean test, which was statistically significant (*p* = 0.048). PSN: pseudo-spontaneous nystagmus.

The bow and lean test was performed in a total of 110 patients. Of the 25 patients with PSN, 21 (84%) exhibited nystagmus consistent with the lean test. Specifically, of 7 cases with canalolithiasis, 5 (71.4%) exhibited nystagmus away from the lesion; of 18 cases of cupulolithiasis, 16 (88.9%) exhibited nystagmus towards the lesion. By contrast, of the 25 patients with PSN, 4 (16%) exhibited nystagmus consistent with the bow test. Specifically, of 7 cases with canalolithiasis, 2 (28.5%) exhibited nystagmus towards the lesion; of 18 cases of cupulolithiasis, 2 (11.1%) exhibited nystagmus away from the lesion. Among 110 patients who underwent both the bow and lean test, all 23 patients with PSN (100%) showed nystagmus, whereas 13 of 87 patients without PSN (14.9%) did not show nystagmus (*p* = 0.048) (Fig 2). Thus, PSN was triggered in 23 of 97 patients (23.7%) who showed nystagmus in the bow and lean test. Of the LC-BPPV patients who underwent the caloric test, six cases showed PSN. Among these, two had canal paresis, which was not related to the direction of PSN. Even without PSN, there were three cases with canal paresis, and this was not related to the direction of PSN. The number of times the barbecue maneuver was performed until the end of treatment was 1.4 ± 0.7 in patients with PSN and 1.4 ± 0.9 in patients without PSN. The difference in treatment outcome between the two groups was not statistically significant (*p* = 0.976) (Table 1) (see S1 Dataset).

## Discussion

The incidence of PSN in LC-BPPV varies widely in the literature, and is reported to range from 15–76% [7, 13–16]. In this study, the incidence of PSN was 19.1%, which is similar to the incidence presented in some studies and relatively low compared to others. The reason for this difference might be related to several factors. First, different methods have been used to study nystagmus. The majority of studies that have investigated PSN have used video-oculography goggles and electronystagmography (ENG) for analyses of nystagmus. ENG shows a relatively high sensitivity compared to video-oculography goggles, which may have influenced the reported incidence of PSN. Moreover, the anatomy of the skull and semicircular canals varies according to race, which should be considered because reports citing relatively low PSN incidence have been conducted mostly in Asia. In addition, to provoke PSN in LC-BPPV, stimulation of the cupula of the LC by any mechanism is necessary, but the incidence of PSN may vary depending on the time of the visit and the degree of symptoms. Our study targeted patients who visited an outpatient clinic, and considering that the severity of symptoms of patients who visit the emergency room is more serious, it is presumed that these factors at least partially explain our lower incidence than previous studies.

The mechanism of PSN in LC-BPPV remains controversial. Von Brevern et al. suggested that the probable mechanism of PSN was the functional plugging of the LC with otoconial debris, resulting in permanent ampullofugal deflection of the cupula caused by negative endolymph pressure between the cupula and the plug [17]. However, this theory has limitations that cannot explain PSN induced in both directions. Recently, the most commonly accepted mechanism, which was first introduced by Asprella-Libonati, is that PSN in LC-PBBV is due to the 30° inclination of the LC with respect to the horizontal plane [13]. When the patient is in the sitting position with his or her head perpendicular to the horizontal plane, the anterior part of the LC is upwardly inclined by approximately 30°. It behaves as an inclined plane, where otoliths can slowly move down via gravity. This theory can explain the very low incidence of involvement of the vertical canals, as in our study, where there is anatomical difficulty triggering the endolymphatic flow in the vertical canal in the sitting position. In addition, it suggests the possibility of PSN in both directions depending on the location of the otoconial debris in the semicircular canal.

In the present study, the incidence of PSN in the canalolithiasis and cupulolithiasis groups did not differ. That is, the LC-BPPV subtype could not be estimated by the presence or absence of PSN. The direction of PSN was predominantly on the healthy side in canalolithiasis cases and on the lesion side in cupulolithiasis cases. This is in accordance with the results of the lean test, because the physiological mechanism of PSN is identical to that of the lean test, in that the LC is inclined approximately 30 degrees from the pitch plane in the sitting position [12]. In this study, there were also cases with PSN on the opposite side. Asprella-Libonati described PSN toward the lesion side in canalolithiasis as the apogeotropic form, due to otoconial debris of the canal close to the cupula side floating downward toward the ampulla (ampullopetal flow) by both gravity and brisk deceleration [13]. There have been few reports of PSN toward the healthy side in cupulolithiasis, and the possibility of superimposed canal paresis has been suggested as the cause [18]. However, because the caloric test is not routinely recommended for patients diagnosed with BPPV, the number of trials is small and thus the reliability is somewhat lower. In our study, the estimated direction of spontaneous nystagmus from canal paresis was not matched to the direction of PSN, although the number of cases was small. Moreover, all cases showing PSN toward the opposite side showed a direction of nystagmus consistent with the bow test, which differed from the estimated side considering the affected ear and subtypes. We assumed that patients with LC-BPPV tend to set their head in the null position in the pitch plane to decrease their symptoms. Therefore, some patients might have had a flexed head position at the time of test although they were requested to sit straight. When the direction of PSN coincided with the direction of the bow test, canalolithiasis in the direction of PSN or cupulolithiasis in the opposite direction was observed. On the other hand, when the direction of PSN matched the lean test, canalolithiasis was in the opposite direction or cupulolithiasis was in the same direction of PSN (Table 3). As mentioned above, the majority of our patients showed PSN toward the contralateral side in canalolithiasis and toward the ipsilateral side in cupulolithiasis, consistent with nystagmus on the lean test. Therefore, in most cases, it is expected that nystagmus matches the results of the lean test, as in the current study.

**Table 3:** Relationship between direction of PSN and the bow and lean test in LC-BPPV patients.

PSN	Bow test	Lean test	Suspected location of otoconia
RB	RB	LB	Right canalolithiasis
			Left cupulolithiasis
RB	LB	RB	Left canalolithiasis
			Right cupulolithiasis
LB	RB	LB	Right canalolithiasis
			Left cupulolithiasis
LB	LB	RB	Left canalolithiasis
			Right cupulolithiasis

PSN: pseudo-spontaneous nystagmus; LC-BPPV: lateral semicircular canal benign paroxysmal positional vertigo; RB: right-beating nystagmus; LB: left-beating nystagmus.

The bow and lean test was not positive in all BPPV patients, but it was positive in all patients with PSN. This may be because both the bow and lean and PSN are tests in which nystagmus is triggered by stimulation in the pitch plane. The LC is ideally stimulated by head motion in the yaw plane. It is conceivable that the increased sensitivity of the cupula or increased mobility of the otolith influenced the LC to respond to the motion in the pitch plane. In addition, the sitting position in which PSN is induced has a lesser degree of head tilt than the bow and lean test, so the stimulus that induces PSN could also trigger nystagmus in the bow and lean test. On the contrary, the fact that PSN is less likely to be triggered in patients with a positive bow and lean test reaffirmed our hypothesis. Therefore, we suggest that the bow and lean test should be performed simultaneously in LC-BPPV patients with PSN. Another benefit of this test in patients with PSN is that it provides important information to rule out vestibulopathy. In patients with spontaneous nystagmus, other vestibular disorders such as vestibular neuritis, labyrinthitis, and Ménière’s disease should be considered. In most patients who performed the bow and lean test in our study, the direction of nystagmus changed, or in rare cases, nystagmus disappeared. Given that patients with vestibulopathy show constant spontaneous nystagmus regardless of positional changes, this may help obtain a correct diagnosis.

In the present study, treatment outcome did not differ according to the presence of PSN, and there were no significant differences according to the subtype of BPPV. These results are consistent with some previous studies [8, 10]; however, other studies have reported that PSN adversely affects treatment outcomes [14, 18]. Further studies with larger sample sizes are warranted to reach accurate conclusions. The limitations of our study include those due to a retrospective study design. Because the incidence of PSN was low in LC-BPPV patients, the sample size of the PSN-positive group was relatively small. Moreover, because various other vestibular function tests were not routinely performed for the diagnosis of BPPV, it was difficult to find factors that correlated with PSN. Therefore, prospective investigations concerning the occurrence and characteristics of PSN in patients with LC-BPPV are needed.

## Conclusion

PSN was observed in patients with LC-BPPV. Considering the anatomical position, the pattern of nystagmus in most patients was similar to that in the lean test. Moreover, depending on the location of the otolith or the head position, some patients showed the same results in the bow test. In LC-BPPV, nystagmus was most likely induced by motion in the yaw plane while lying down, but it could also be seen during motion in the pitch plane in the sitting position. The more strongly the cupula is deviated by this motion, the higher the probability of PSN induction.

## Supporting information

S1 Dataset Detailed information for the included LC-BPPV patients (n = 131). LC-BPPV: lateral semicircular canal benign paroxysmal positional vertigo.
(XLSX)
